# Challenges with patient management of osteoarthritis during the COVID-19 pandemic: review

**DOI:** 10.1097/MS9.0000000000000978

**Published:** 2023-06-21

**Authors:** Tarika Deepak Patel, Olivia Campos Coiado

**Affiliations:** a Carle Illinois College of Medicine; b Department of Biomedical and Translational Sciences, Carle Illinois College of Medicine, University of Illinois Urbana-Champaign, Champaign, IL

**Keywords:** Arthroplasty, COVID-19, joint, management, osteoarthritis, public health

## Abstract

Osteoarthritis is a growing public health concern, affecting millions of people worldwide. With progressively worsening joint function and pain, management of osteoarthritis is important to ensure high quality of life for patients. Treatment includes a combination of pharmacologic agents and non-pharmacologic methods such as exercise and physical therapy. However, if multiple treatments fail to improve symptoms, joint replacement surgery is the final course of action. When the new coronavirus, SARS-CoV-2 (COVID-19), was declared a pandemic, all aspects of osteoarthritis treatment become affected. Due to increased public health measures, non-pharmacologic modalities and elective surgeries became limited in accessibility. Additionally, there were concerns about the interaction of current medications for osteoarthritis with the virus. As a result of limited options for treatment and quality of life of patients was negatively impacted, especially in those with severe osteoarthritis. Furthermore, a backlog of joint replacement surgeries was created which could take up to several months or years to address. In this review, we describe the impact COVID-19 had on osteoarthritis management as well as tactics to deal with the large caseload of surgeries as operative rooms begin to re-open for elective surgeries.

## Introduction

Highlights

- Telehealth provides successful physical therapy and exercise intervention during the pandemic.
- Pharmacologic management of osteoarthritis should be continued during the pandemic.
- Increase patient access to non-operative management options to limit opioid use and progression of osteoarthritis symptoms.
- Encourage interprofessional collaboration to address influx of new surgical cases and backlog of cancelled cases.

Considered a public health concern, osteoarthritis (OA) is an incapacitating disease often associated with long-term psychological and physical sequelae^1^. While disease progression occurs over several years, individuals ultimately develop joint failure with pain and disability^1^.

Symptomatic OA affects millions of people worldwide^2^. The goals of OA treatment are to minimize both pain and functional loss^3^. Disease management includes a combination of non-pharmacologic and pharmacologic therapies^3^. Variation in patient response to treatment is observed as there is a large component of trial and error in selecting the most effective treatment strategies^3^.

In March 2020, the 2019 SARAS-CoV-2 (COVID-19) was declared a pandemic. With increasing hospitalizations, there were critical shortages in medical equipment. Thus, public health measures were put into place to decrease the spread of the virus^4^. These recommendations impacted a large cohort of patients with OA^4^. In this review we aim to understand the effects COVID-19 had on OA management and how such changes impacted patients’ outcomes. We describe a simple literature review with updated recommendations for OA management, triage strategies for arthroplasty, pathways for resuming elective surgery during a pandemic, and valuable innovations.

## Methods

PubMed search engine and Google Scholar were used to compile a total of 46 studies for this review. The search strategy for compiling the literature included several methods as outlined in Supplemental Figure 1, Supplemental Digital Content 1, http://links.lww.com/MS9/A167. Using PubMed, reports were initially screened by reading the title and abstract for relevance to osteoarthritis management and arthroplasty in the setting of the pandemic. Studies were then filtered based on article type to exclude case reports, books, and reports not in English. Google Scholar searches were filtered to only include reports that have the search terms in the title. The process for filtering, inclusion, and exclusion was the same. Multiple search phrases and search engines were used to reduce bias and allow for increased inclusion of pertinent studies.

## Pre-COVID osteoarthritis management

OA management requires a versatile approach that includes non-pharmacologic therapies, pharmacologic therapies, and surgery as a last resort. Weight loss can be a significant intervention for those who are overweight; every pound lost can decrease the load on the knee by 3–6 fold^3^. Engagement in low-impact aerobic exercise has been recommended^5^. The addition of formal physical therapy (PT) can assist patients in using assistive devices appropriately while also instructing them on proper exercise techniques^3^.

Acetaminophen and non-steroidal anti-inflammatory drugs (NSAIDs) are initial choices for pharmacologic treatment with NSAIDs being first-line^3,5^. Other agents include capsaicin and tramadol for refractory symptoms^5^. and intra-articular corticosteroids or hyaluronic acid injections^3^. Lastly, opioids and duloxetine can be used in those without adequate response to the previous pharmacologic treatments and who may not be candidates for surgery^3,5^.

Surgical joint replacement is next in line in patients specifically with knee or hip OA who have failed multiple non-pharmacologic and pharmacologic treatment modalities^3^. Joint replacements can provide pain relief and increase functionality, and failure rates are low^3^. A comparison of osteoarthritis management before and during COVID-19 is summarized in Table 1.

**Table 1:** Comparison of osteoarthritis management recommendations before and during COVID-19 pandemic

Method of OA management	Pre-COVID	During COVID
Non-pharmacologic		
Exercise and weight loss	No restriction on environment Regular low-impact aerobic exercise Regular in-person follow-ups with physician	Restriction on environment Regular at-home exercise with a goal of a 10-min walk at a brisk pace each day Regular virtual follow-up or continuous monitoring with wearable device
Physical therapy	Regular in-person-based Help with assistive devices Regular in-person follow-ups with physician	Home-based and telehealth-based Regular virtual follow-up
Pharmacologic		
NSAID	First-line medication for symptom management	No obvious impact on developing COVID-19 outcomes or susceptibility Encourage as first-line option for symptom management
Acetaminophen	Symptom management Alternative for those not tolerable to NSAIDs	No obvious impact on developing COVID-19 outcomes or susceptibility Encourage as option for symptom management
Corticosteroids	Intra-articular injections for severe, refractory pain	Limited appointment availability for injections No increased severe risk of infection or major complications Encourage steroid injections for severe pain Monitor for complications with immunosuppression
Opioids	Indicated for those with poor response to other interventions Monitor for immunosuppression	Weak opiates with no immunosuppression are preferred with monitoring Strong opiates require careful monitoring Discontinuation not supported
Surgical		
Joint arthroplasty	Last line after multiple failed interventions No limitations regarding scheduling or postoperative follow-up	Limited accessibility to joint arthroplasty Increase access to non-operative management options during increased wait periods Monitor postoperative patients remotely or with trained home familial caregiver Surgical protocol that emphasizes a COVID-19-protected ward/zone, shortened hospitalizations, and increased use of telerehabilitation Delay arthroplasty for at least 3 months after ICS injection Triage and prioritization with EQ-5D-3L and RAPT scores with high considerations for PPF and postoperative sepsis

EQ-5D-3L, European quality of life five dimension; ICS, intraarticular corticosteroid; NSAID, non-steroidal anti-inflammatory drug; OA, osteoarthritis; PPF, peri-prosthetic fracture; RAPT, Risk Assessment and Prediction Tool.

## Non-operative management of osteoarthritis during COVID-19

### Non-Pharmacologic management

General practitioners are often the first medical professionals to treat musculoskeletal complaints. However, there was a 50% reduction in general practitioners consultations for hip/knee OA during the first wave of the pandemic^6^. Additionally, data indicated a significant drop in PT referrals placed in 2020 (1972 referrals) compared with 2019 (1614 referrals) for patients with OA^7^. Losing access to public transit and increasing fears of healthcare facilities could have decreased willingness to attend appointments^7^. A continuation of these trends can cause worsening mobility and fragility among OA patients and consequently more requests for arthroplasty surgery^6^.

Telehealth has become a feasible option for delivering high-quality healthcare. The use of telehealth for PT has favourable efficacy in improving outcomes in patients with orthopaedic conditions^8^. One study showed that telephone-based PT led to improvements in pain, joint stiffness, and physical function in patients with knee OA^9^. In fact, telerehabilitation could be comparable with in-person rehabilitation^10^. Similarly, telehealth-based exercise achieved the same effects of traditional face-to-face treatment for OA management, and pain was significantly alleviated^11^. 58.3% and 32% of patients were extremely satisfied and satisfied, respectively, with their telemedicine visit^12^. Thus, telehealth for PT and rehabilitation appears to be a suitable method of care for patients with OA during the pandemic.

Low-impact aerobic exercises like walking, biking, or resistance training are the most effective for managing OA pain^13^. Most patients became less active during the pandemic, causing an increase in pain and loss of joint function, demonstrating the importance exercise during the lockdown^14^. Walking at a brisk pace (100 steps/min) for at least 10 min a day is the minimum amount of exercise recommended for adults with OA^15^. Most importantly, physicians and physical therapists should regularly contact patients by phone or virtual session to monitor the clinical status of patients^13^. Wearable devices like a Fitbit can help healthcare workers to monitor changes in physical activity and specifically target individuals who may need additional reinforcement^16^. A key strength of this approach is real-time documentation instead of relying on self-reported data^16^. For non-pharmacologic management, it is recommended that physicians encourage at-home physical therapy via telemedicine and at-home exercises along with continuous monitoring with a wearable device or regular virtual follow-up.

### Pharmacologic management

COVID-19 also impacted pharmacologic management of OA due to concerns about interactions with the virus. NSAID use increased COVID-19 complications such as complicated pneumonia and pleural effusions^17^. Acetaminophen does not increase infectious risk but can underestimate the severity of the disease and delay the presentation of symptoms which leads to a delayed diagnosis and worse prognosis^17,18^. However, a comparator cohort study compared the risk of acquiring COVID-19 when using NSAIDs, ibuprofen, or paracetamol, all of which are common analgesics used for OA pain^19^. Calibrated hazard ratios revealed no significant differential risk of COVID-19 outcomes or hospitalizations in users of ibuprofen versus any other analgesic classes^19^. It is recommended to reassure the community that analgesia use is safe with regards to infection susceptibility and encourage continued use for pain benefit.

Corticosteroids are given intra-articularly for OA symptoms due to their powerful anti-inflammatory nature. No evidence exists suggesting that a net benefit or detriment is received from corticosteroids in COVID-19 patients^17^. A systematic review found that there was a low likelihood of contracting COVID-19 after injection of steroids^20^. The population had no increased risk of severe infection and no major complications were reported^20^. Their use during COVID-19 infection must be carefully monitored for potential complications with immunosuppression^18^. It is recommended that steroid injections be used for severe pain management as it can improve pain outcomes and reduce the need for immediate surgery.

Opiates have become a valuable treatment option when other analgesics are ineffective. When needed, weak opioids with no immunosuppressive activity like tramadol are preferred, but for patients on strong opioids, careful surveillance is needed for the potential of abuse/misuse and respiratory depression^17,18^.

## Arthroplasty during COVID-19

To combat the spread of COVID-19, the American College of Surgeons recommended postponing or cancelling elective procedures to preserve medical resources and minimize the burden on the healthcare system^21^. If all orthopaedic physicians followed these guidelines, 30 002 total hip arthroplasty (THA) and total knee arthroplasties (TKA) would be cancelled each week in 2020^21^. In 2020, an estimated 526 000 to 538 000 total joint arthroplasties (TJA) were performed, representing a 46.5–47.7% decrease in volume nationwide^22^. It would take 9–35 months to reschedule cancelled surgeries^23^. This trend was observed worldwide as other countries such as Denmark, the Netherlands, Sweden, Norway, Chile, and Germany saw a decline in the number of arthroplasties performed^24–28^. Consequently, patients had continued deterioration in pain, joint function, and physical function^14^. The long wait for surgeries caused an increase in opioid-based analgesia for the exacerbation of symptoms^29^. However, the reason for increasing opioid-based pain control is lacking^29,30^. While patients wait for surgery, it is recommended that healthcare professionals adapt delivery of care to meet unmet demands which can include increasing patient access to non-operative management options as previously mentioned.

Post-arthroplasty follow-up and rehabilitative care were restricted; however, there are mixed results regarding the effect on clinical outcomes. One study saw a significant correlation between inferior clinical outcomes at 6 months and missed physiotherapy^31^. Another study saw that despite decreased care access and post-surgical PT compared with pre-pandemic patients, 1-year clinical outcomes were equivalent^32^. This highlights that there may be an over-emphasis on in-person follow-up and PT for post-arthroplasty patients and requires future exploration in the future. It is recommended that postoperative patients continue light home PT to prevent deconditioning. If available, trained home caregivers (spouse or family member) can effectively and reliably score the function of the knee of patients who had TKA and assist monitoring function of the replaced joint^33^.

## Resuming elective orthopaedic surgeries

Restarting elective joint replacements is an important milestone in the recovery from the pandemic, but it does come with significant obstacles. Only a few states published guidelines specific to orthopaedic surgery during the pandemic, leaving hospitals and surgeons responsible for balancing the benefit of surgery with the costs to public health^34,35^.

Patient safety is important when re-establishing arthroplasty surgeries. Informed consent should include clarifications about high risk of virus transmission, limitations in care, and the potential need for ventilation^35^. With the multilevel approach, five levels were described to minimize the risk of exposure to the virus through symptom screening, self-questionnaires, temperature checkpoints and masking, social distancing, and minimal staff contact^36^. Several hospitals also developed their own surgical protocol to maintain safety. An institution in India included a protocol where if a pre-anaesthetic check-up determined patients as fit for operation, patients were required to home quarantine for 14 days and have a negative COVID test before surgery would be scheduled^37^. A dedicated orthopaedic ‘ring-fenced ward’ strategy provided a COVID-19-protected environment with limited access and a focus on patient and staff safety^37^. Similarly, some countries like England and Scotland implemented strict pathways/zones that a patient may enter based on their designation as green (COVID-light) or red (confirmed COVID). Adherence to these strict guidelines allowed for elective orthopaedic services during the pandemic while avoiding postoperative COVID-related complications^38^. A second group focused on early discharge. During recovery after surgery, emphasis was placed on intensive PT with earlier ambulation and frequent session. Patients were discharged over 1 day earlier compared with pre-pandemic patients with improvements in clinical outcomes similar to pre-pandemic patients^39^. Additionally, unsafe timeframes for rescheduled arthroplasty needs to be considered. Due to steroids’ immunomodulatory effect, prosthetic joint infection (PJI) is a concern^20^. A systematic review found that there were increased odds of PJI when intra-articular corticosteroids were administered 12 months before arthroplasty; odds of PJI increased significantly if steroids were given within 3 months before surgery^20^.

As the incidence of COVID-19 begins to decline, elective orthopaedic procedures can slowly be reinstated, and the backlog of surgical cases can be addressed. However, guidelines to reduce the spread of the infection should remain in hospitals to protect patients and staff and decrease postoperative and COVID-19 complications. Additionally, a protocol should leverage a COVID-19-protected ward/zone and shortened hospitalizations. It is also recommended that patients delay arthroplasty for at least 3 months after intraarticular corticosteroid injection.

## Strategies for arthroplasty triage and prioritization

Orthopaedic surgeries during the pandemic and re-opening phase require careful case prioritization and triage management to assure patient safety while addressing the increasing backlog of surgical cases. Engineers translated strategies used to address backlogs in computer servers to healthcare which includes scale-up of resources, queuing, buffering, and dropping^40^. Scaling up implies increasing surgical throughput through increased utilization of dedicated teams for orthopaedic care that can enhance throughput^40^. Queuing and buffering involve prioritizing some elective surgical patients based on urgency and severity^40^. Dropping, entails redefining surgical indications to render a subset of patients ineligible for surgery based on comorbidities, but could generate bias^40^. Other studies applied these strategies.

Members of the British Association for Surgery of the Knee (BASK) working group were given TKA clinical scenarios. Primary arthroplasty, revision procedures, aseptic loosening without risk of collapse were considered low priority and could be delayed for 3 months or longer if there is severe pain or decline in mobility^41^. Early debridement, antibiotic, and implant retention for sepsis and peri-prosthetic fracture (PPF) were considered the most urgent and required surgery within 24 h^41^. Similarly, after surveying members from the European Hip Society, PPFs and acute infections were considered as the number one priority^42^. For more objective assessments of prioritization, preoperative and 1-year postoperative European quality of life five dimension (EQ-5D-3L) was measured in patients awaiting TKA and THA. Patients with an initial low EQ-5D-3L (−0.239 to 0.487) had significantly greater gains postoperatively along with greater improvements in Oxford hip and knee scores^43^. Thus, a preoperative EQ-5D-3L cut-off of less than 0.487 could assist with the prioritization of non-emergent arthroplasties.

Triage for outpatient versus inpatient postoperative recovery also requires careful consideration due to due to limited hospital occupancy. The Risk Assessment and Prediction Tool (RAPT) could predict patient discharge disposition following arthroplasty. A group studied its predictive ability and found that scores varied significantly between same-day discharge, next-day discharge, and inpatient stay; scores were 10, 9, and 8, respectively^44^. However, the pandemic was seen to correlate with significant decreases in mean RAPT scores for outpatient and inpatient designations^44^. RAPT scores can be utilized, however, comorbidity burden and other considerations must be identified given a more strict threshold to qualify for inpatient recovery during the pandemic. Triaging patients post-TJA for hospital or video consultation can also help to manage patient flow. Expert panels defined having moderate or severe pain and using 2 crutches as the triage tool criteria^45^. Using the triage tool, around 70% of THA patients could safely have video consultations 6 weeks postoperatively^45^.

During the pandemic, triage and prioritization of orthopaedic surgeries can help reduce extra burden in time and costs while still addressing the growing waiting list. It is recommended to prioritize PPFs and sepsis procedures. Elective joint arthroplasty can be postponed until at least 3 months later with prioritization with the EQ-5D-3L criteria. Additionally, triage tools can be helpful to limit inpatient recovery after surgery and can continue to be used post-pandemic to maintain efficient patient flow.

## Pandemic-inspired innovative solutions for multifaceted osteoarthritis management

The devastating impact of COVID necessitates novel solutions in multiple areas to support continued therapy for osteoarthritis. Telehealth became a popular tool; thus specific training is required to effectively provide care. The Physiotherapy Exercise and Physical Activity for Knee Osteoarthritis (PEAK) e-learning modules trains physiotherapists in virtual OA management. Learners demonstrated a 64–74% increase in confidence with videoconferencing and 59.0% described the e-learning program as extremely useful^46^. There is acceptability of an e-learning program for effectively training therapists on telehealth-based OA management^47^.

Taking advantage of advancing technology can provide new approaches to rehabilitation like virtual reality-based physical therapy. Patients had improved pain and function of the joint when they exercised using Nintendo Wii Fit gaming stations or virtual reality glasses with a smartphone-based game^48^. Artificial intelligence has the potential to rapidly and correctly prioritize patients for arthroplasty. The artificial intelligence to Revolutionize the Patient Care Pathway in Hip and Knee Arthroplasty (ARCHERY) project focuses on using machine learning to determine demographic, clinical, and imaging characteristics that influence patients selection for arthroplasty and to develop a predictive model to guide arthroplasty referral pathways^49^. Anticipated findings in the context of COVID-19 include the development of an algorithm that will provide improved quality care and reduced administrative burden^49^.

Lastly, a medical group created a “frugal” containment sheet to minimize aerosol and splatter dispersion during arthroplasty. The use of the containment sheet contained and reduced the dispersion of particles in a cost-effective matter^50^. In the absence of full protective gear, as seen during the pandemic, this innovation can mitigate risks to patients and staff under public health constraints^50^. The pandemic encouraged collaboration and innovation among physicians and researchers to create novel solutions for the challenges the healthcare system faced.

## Limitations

PubMed and Google Scholar were the two primary search engines used to generate this review. Publications and journals not PubMed indexed may not have been included and is one source of bias and limitation to the study. Specific search criteria allowed for the inclusion of relevant papers. Overall, new research in this area is continuously being published, thus there is a continuous flow of new information.

## Conclusion

The declaration of the 2019 SARS-CoV-2 (COVID-19) as a pandemic led to detrimental effects on the healthcare system. Many states implemented lockdowns and hospitals postponed elective surgeries to re-direct resources for COVID-19 patients. As a result, patients with chronic conditions, such as osteoarthritis, face challenges with all modalities of treatment.

While non-pharmacologic treatment became more difficult to access in person, the utilization of telehealth and other virtual strategies made it possible to continue exercise and PT safely. There were concerns regarding interactions between OA pharmacology and COVID-19; however, there is no evidence of detrimental effects or increase in viral incidence. TJAs also became challenging to receive as most cases were cancelled or postponed until further notice. Thus, healthcare providers are encouraged to adjust care to increase non-operative options while waiting for surgery.

As hospitals begin to re-open elective surgeries, a backlog of cases has accumulated. Thus, the pandemic has challenged the healthcare system to develop creative ideas for safe arthroplasties during the pandemic and new tools for arthroplasty triage and prioritization that can control patient flow and encourage staff safety. Many of these developments can continue to be refined and applied after the end of the pandemic as it helps make osteoarthritis and arthroplasty management more streamlined and efficient in a growing deficit of providers. This is an area for future research as it can dramatically assist healthcare workers.

## Ethical approval

NA.

## Consent

NA.

## Source of funding

None.

## Author contribution

T.P. and O.C.C. conceived of the presented idea. T.P. searched the manuscripts for this literature review, verified the methods and wrote the manuscript. O.C.C. reviewed, edited and supervised the findings of this manuscript.

## Conflicts of interest disclosure

None.

## Research registration unique identifying number (UIN)

NA.

## Guarantor

Tarika Patel and Olivia Campos Coiado.

## Data availability statement

NA.

## Provenance and peer review

Not commissioned, externally peer-reviewed.

## Supplementary Material
