# Supplementary figures and images for: Challenges with patient management of osteoarthritis during the COVID-19 pandemic: review
Source: Ann Med Surg (Lond). 2023 Jun 21;85(8):3925–30. doi: 10.1097/MS9.0000000000000978 (PMC10406077; doi:10.1097/MS9.0000000000000978)

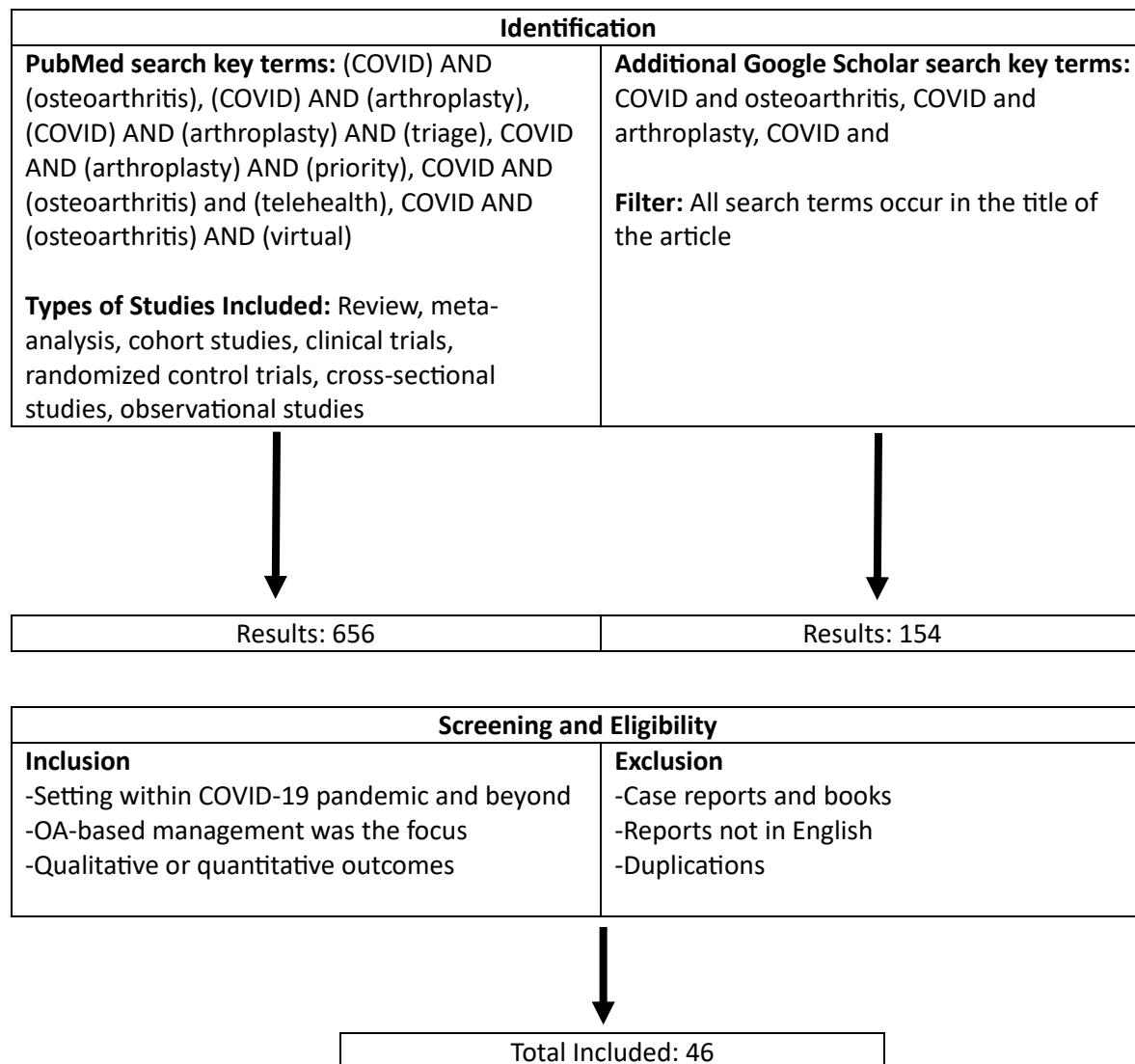

**Supplemental Figure 1:** Research and search method

Supplement: Supplementary file 1 [file ms9-85-3925-s001.pdf]
